# Benefits in the Macrophage Response Due to Graphene Oxide Reduction by Thermal Treatment

**DOI:** 10.3390/ijms22136701

**Published:** 2021-06-22

**Authors:** Mónica Cicuéndez, Laura Casarrubios, Nathalie Barroca, Daniela Silva, María José Feito, Rosalía Diez-Orejas, Paula A. A. P. Marques, María Teresa Portolés

**Affiliations:** 1 Departamento de Bioquímica y Biología Molecular, Facultad de Ciencias Químicas, Universidad Complutense de Madrid, Instituto de Investigación Sanitaria del Hospital Clínico San Carlos (IdISSC), 28040 Madrid, Spain; mcicuendez@ucm.es (M.C.); laura.casarrubios.molina@gmail.com (L.C.); mjfeito@ucm.es (M.J.F.); 2 Center for Mechanical Technology & Automation (TEMA), Mechanical Engineering Department, University of Aveiro, 3810-193 Aveiro, Portugal; nbarroca@ua.pt (N.B.); danielas@ua.pt (D.S.); 3 Departamento de Microbiología y Parasitología, Facultad de Farmacia, Universidad Complutense de Madrid, 28040 Madrid, Spain; rdiezore@ucm.es; 4 CIBER de Bioingeniería, Biomateriales y Nanomedicina, 28040 Madrid, Spain

**Keywords:** graphene oxide, reduced graphene oxide, macrophage, cytokine, immune response

## Abstract

Graphene and its derivatives are very promising nanomaterials for biomedical applications and are proving to be very useful for the preparation of scaffolds for tissue repair. The response of immune cells to these graphene-based materials (GBM) appears to be critical in promoting regeneration, thus, the study of this response is essential before they are used to prepare any type of scaffold. Another relevant factor is the variability of the GBM surface chemistry, namely the type and quantity of oxygen functional groups, which may have an important effect on cell behavior. The response of RAW-264.7 macrophages to graphene oxide (GO) and two types of reduced GO, rGO15 and rGO30, obtained after vacuum-assisted thermal treatment of 15 and 30 min, respectively, was evaluated by analyzing the uptake of these nanostructures, the intracellular content of reactive oxygen species, and specific markers of the proinflammatory M1 phenotype, such as CD80 expression and secretion of inflammatory cytokines TNF-α and IL-6. Our results demonstrate that GO reduction resulted in a decrease of both oxidative stress and proinflammatory cytokine secretion, significantly improving its biocompatibility and potential for the preparation of 3D scaffolds able of triggering the appropriate immune response for tissue regeneration.

## 1. Introduction

Due to their unique physical and chemical properties [1], graphene and its derivatives are very promising nanomaterials for a wide range of biomedical applications [2], such as biosensing [3], bioimaging [4], drug delivery [5], and photothermal therapy [6]. In the last decade, graphene oxide (GO) and reduced graphene oxide (rGO) have proven to be very useful for the preparation of scaffolds for tissue repair, capable of acting as support for growing cells in a suitable microenvironment [7,8,9]. GO is electrically insulating, owing to its disrupted sp^2^ bonding network due to the presence of oxygen functional groups, such as hydroxyl and epoxy functional groups on the basal plane and carbonyl/carboxylic acids groups on the plane edges [10]. However, rGO derives from the reduction of GO, which restores the π network imparting electrical conductivity, a key feature when designing electroconductive devices [11]. There are different types of reduction processes, such as chemical, thermal, microwave, and UV light reduction, with different advantages and disadvantages related to the reduction degree, improvement of material properties, toxicity, and economic cost, among other aspects [7]. Several factors have been identified as the culprits for GO and rGO cytotoxicity, such as dose, lateral size, and surface charge [12]. Another relevant factor is the variability of GO surface chemistry, namely the type and quantity of oxygen functional groups, which may have an important effect on cell behavior [13]. Although there is a low number of studies comparing cellular responses to GO and rGO, some authors indicate that rGO is less toxic than GO [14,15], while others suggest that rGO may cause more plasma membrane disruption and oxidative stress than GO [12]. Several studies have pointed out that GO induces an inflammatory response and chronic injury by interfering with the functions of important organs such as the respiratory tract, the central nervous system, and blood components [16]. In this context, the response of immune cells to these graphene derivatives appears to be critical due to macrophage functional plasticity between two extremes, designated as proinflammatory (M1) and reparative (M2) phenotypes [17]. Thus, the balance between M1 and reparative M2 macrophages has been related to the role of this cell type in disease processes and tissue remodeling after injury [18,19]. These macrophage phenotypes are characterized by differences in the expression of distinct cell surface markers and particular genes and the secretion of different cytokines, chemokines, and enzymes that allow them to respond to changes in their microenvironment [20,21].

The translation of graphene derivatives to the medical market may rely on their use as single-component devices or their incorporation into natural or synthetic matrices depending on the targeted application. Regardless of the strategy, the fate of soluble GO or rGO from medical devices should be carefully addressed. Considering the family of carbon nanostructures with potential applications in the biomedical field, graphene and its derivatives are considered less cytotoxic than single- and multiwalled carbon nanotubes (CNTs) and fullerenes [22,23,24]. In the case of carbon nanotubes (CNTs), which have previously received the most attention, it is well-known that length and functionalization can cause very different reactions in cells and that it is critical to distinguish between CNTs in terms of physical and chemical properties [25]. Learning from the CNT literature, it is of huge importance to systematically evaluate the specificities of emerging GBM.

In this work, the response of RAW-264.7 macrophages to different doses of GO and rGO nanostructures with different reduction degrees was evaluated by analyzing the uptake of these nanomaterials, the intracellular content of reactive oxygen species, and specific markers of the proinflammatory M1 phenotype, such as CD80 expression and the secretion of inflammatory cytokines such as TNF-α and IL-6. This comparative study, involving flow cytometry, confocal microscopy, and ELISA methods, highlights the effects of both the degree of GO reduction and the dose delivered on macrophage response.

## 2. Results and Discussion

### 2.1. Structural and Morphological Analysis of GO, rGO15, and rGO30 Nanostructures

The GO reduction process employed in this work consisted of vacuum-assisted thermal treatment at 200 °C. We intended to avoid reduction using chemical solvents that are environmentally nonfriendly, so thermal reduction at low temperatures was performed. Furthermore, our group has previously demonstrated the combined favorable outcome of thermally reduced GO microfibers at 220 °C for 2 h with both neural cells and macrophages [26]. While in this previous study, GO-based bulk constructs were thermally reduced for 2 h, in this work, we aimed at studying the effect of particles to be used as additives in tissue engineering scaffolds and opted for much shorter times of reduction. Additionally, as we targeted biological applications wherein a complete reduction of graphene oxide is not desired, shorter time was preferable.

**Figure 1:**
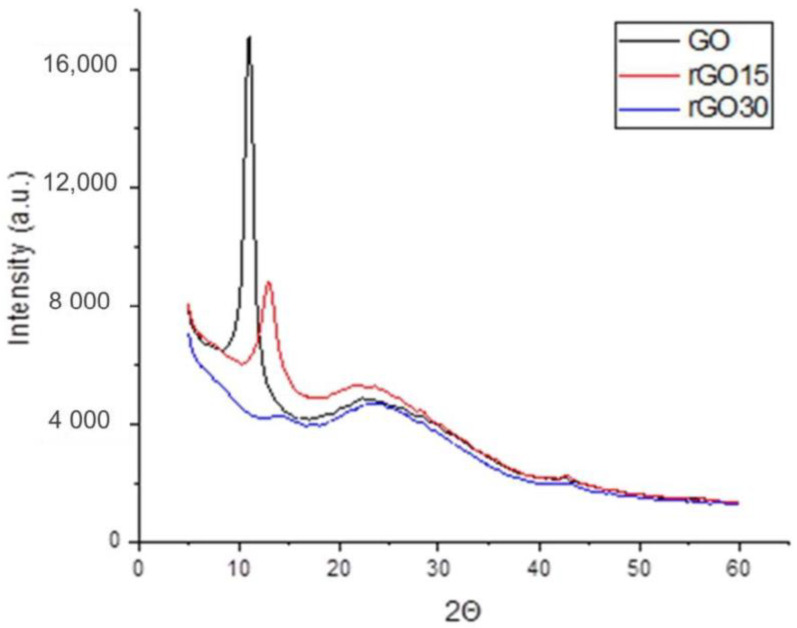
XRD spectra of GO, rGO15, and rGO30.

**Figure 2:**
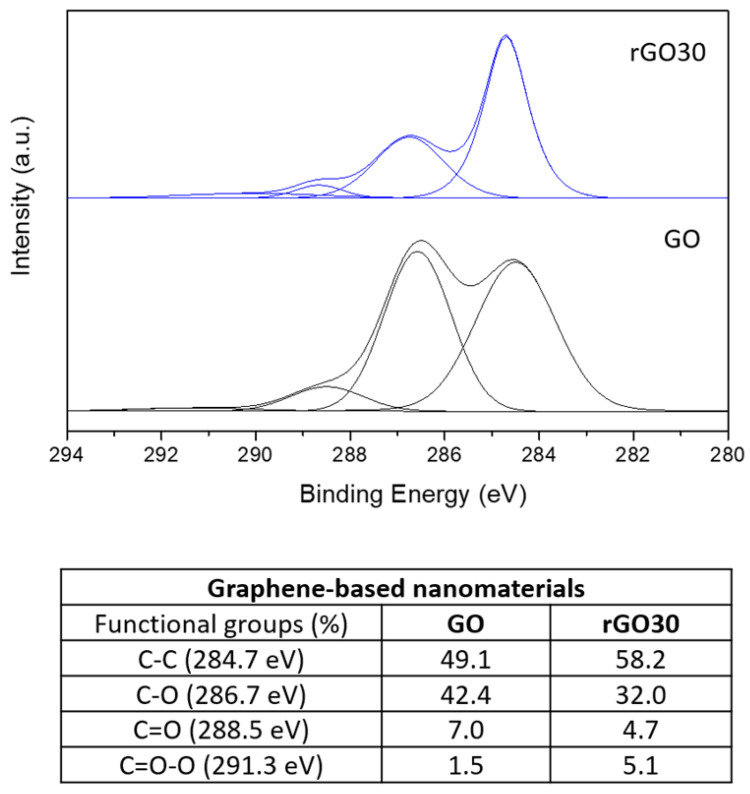
C1s XPS spectra of GO and rGO30, and respective percentages of the present functional groups.

The structural changes of GO after the thermal treatment are shown in the X-ray diffraction (XRD) and X-ray photoelectron spectroscopy (XPS) analyses below (Figure 1 and Figure 2, respectively).

XRD structural analysis (Figure 1) revealed that GO presented a sharp and intense crystalline peak at 2θ = 11.08° that corresponded to the (001) diffraction peak. After the vacuum-assisted thermal reduction at 200 °C for 15 min, the peak was less intense and exhibited a shift to the right (2θ = 12.98°). This could be attributed to water deintercalation, removal of oxygen-containing functional groups, and partial restoration of the sp^2^ network. Moreover, a second broad peak appeared at 2θ = 21.18°, attributed to the (002) plane. A longer thermal reduction time of 30 min induced a further shift of the (001) peak to 2θ = 13.58° and a pronounced broadening due to the partial breakdown of the long-range order of GO [27,28].

These results show that thermal reduction for 15 and 30 min was enough to induce GO reduction. However, the presence of both peaks indicated incomplete reduction. Incomplete reduction of GO is most suited for biomedical applications, as fully reduced GO loses its ability to disperse once most of its oxygen groups are removed, which may make its incorporation into engineered materials for various medical applications difficult. Additionally, residual O-moieties make GO amenable for chemical functionalization, which is valuable for drug delivery [29], cancer therapy [6], and enhancing biocompatibility. 

XPS allowed us to evaluate the deoxygenation more comprehensibly. As seen in the C1s XPS spectrum (Figure 2), GO and rGO30 exhibited the four components relative to carbon atoms in different functional groups: nonoxygenated ring C (284.7 eV), C–O bonds (286.7 eV), carbonyl C=O (288.5 eV), and carboxylate C(O)–O (291.3 eV). 

The peak intensities of the three oxygenated components in rGO30 were significantly lower than those of GO, demonstrating significant deoxygenation during thermal reduction. The majority of the present oxygen moieties are the C–O bonds of epoxy and hydroxyl groups in the basal plane. More oxidized species such as C=O and C=O (O) are sparser. The C=O species come mainly from single ketones that decorate the edges of GO sheets [30] or are bound to the basal plane as carbonyl groups. Quinones are also located at the edges of GO sheets. Regarding the C=O (O) species, these are mostly found at the edges of GO sheets [31,32]. The spectra show that the thermal reduction of GO induced the removal of unstable in-plane oxygen-containing groups. This observation is consistent with previously reported XPS data on low-temperature thermal reduction of GO, predominantly linked to the reduction of hydroxyl and epoxy groups, further shown by the reduction in the percentages of C–O and C=O present in rGO30 [32,33].

**Figure 3:**
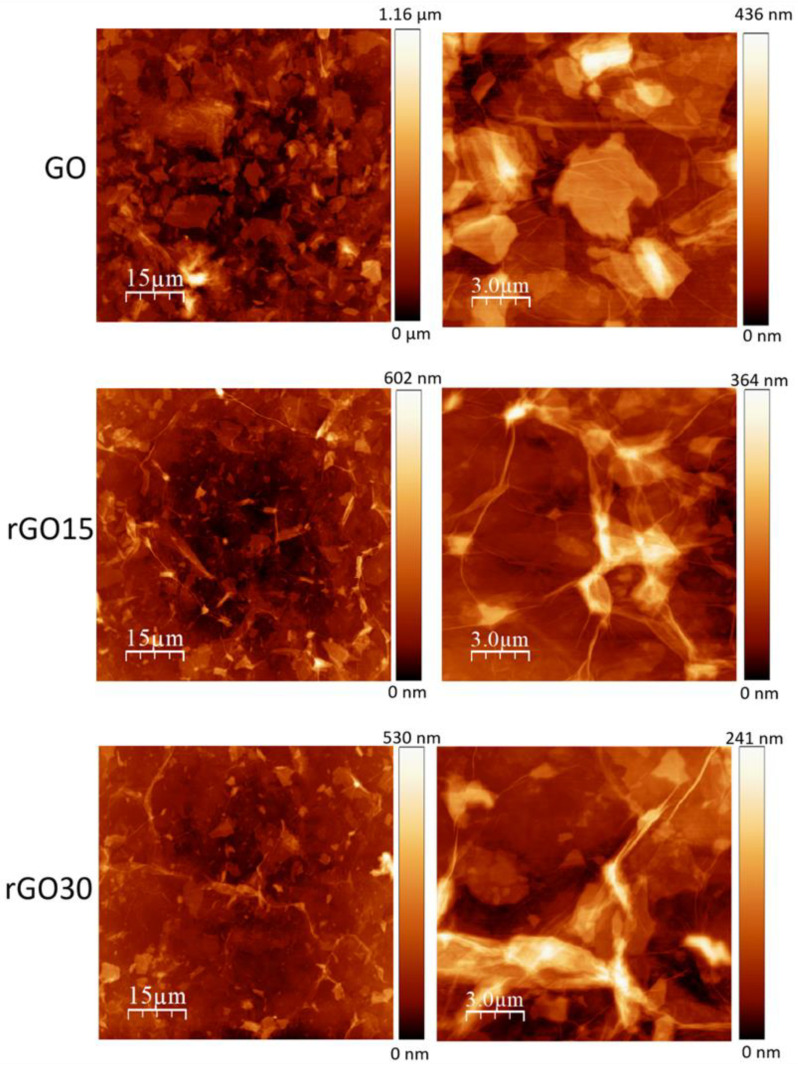
AFM topography images of GO, rGO15, and rGO30.

The morphology dependence of the GO sheets on thermal reduction was further assessed by atomic force microscopy (AFM, Figure 3). 

The morphology of GO and rGO sheets displayed heterogenous size distribution characterized by the presence of large flakes with smaller sheets piling up on top. Additionally, a notable decrease in sheet size upon reduction could be observed. This is consistent with previous observations on reduced GO, even with different reduction methods, such as a chemical one via hydrazine [34]. Additionally, compared to the more flat-like morphology of GO, both rGO15 and rGO30 sheets exhibited some crumpling. These structures are typically the result of the desorption of H_2_O, CO, and CO_2_ and the decomposition of oxygen functional groups that leads to graphene-like sheets with disordered stacking observed upon thermal reduction [27,28,35,36,37].

### 2.2. Uptake of GO, rGO15, and rGO30 by RAW-264.7 Macrophages

**Figure 4:**
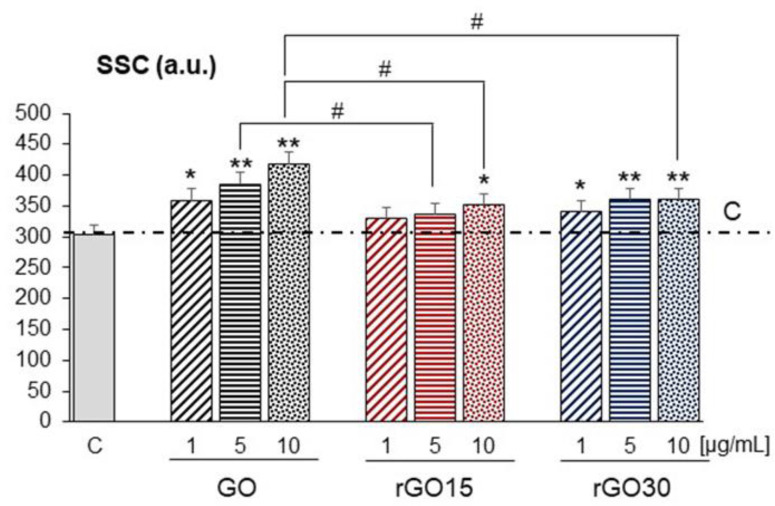
Cell uptake of GO, rGO15, and rGO30 by RAW-264.7 macrophages after 24 h of treatment with 1, 5, and 10 μg/mL of these nanostructures, evaluated by flow cytometry analyzing 90° light scatter (side scatter, SSC). Statistical significance: * *p* < 0.05, ** *p* < 0.01 (compared to control macrophages), # *p* < 0.05 (comparison between nanostructures at the same concentration).

Macrophages are key modulatory and effector cells in the immune response, and their activation influences other components of the immune system in different physiological contexts. These cells perform phagocytic clearance of dead cells during development and adult life and protect the host through innate immunity. Macrophages also play a key role in the removal of nanomaterials or biodegradation products by phagocytosis from scaffolds with potential application in biomedicine, and they are primarily responsible for the uptake and cellular trafficking of nanoparticles in vivo [38]. In this study, cell uptake of different doses of GO, rGO15, and rGO30 nanostructures by RAW-264.7 macrophages was quantified by flow cytometry analyzing 90° light scatter (side scatter, SSC) after 24 h of incubation. This parameter is proportional to the intracellular complexity determined in part by the cellular cytoplasm, mitochondria, and pinocytic vesicles [39]. For this reason, SSC can be used as a measure of the incorporation of these nanostructures inside cells. Figure 4 shows a clear significant dose-dependent increase of the intracellular complexity of macrophages cultured with GO compared to that of the control macrophages. Regarding the results obtained with rGO15, we only observed a significant increase of SSC with macrophages exposed to 10 µg/mL. However, the three assayed doses of rGO30 produced a significant increase in macrophage intracellular complexity compared to that of the control macrophages. Figure 4 also shows the statistical significance among the different nanostructures at the same concentration. Thus, a significant SSC increase (# *p* < 0.05) of macrophages cultured with GO compared to that of rGO15 was observed at the same concentration of 5 µg/mL. Moreover, we also observed a significant increase (# *p* < 0.05) in the intracellular complexity of macrophages exposed to 10 µg/mL of GO compared to that shown by macrophages exposed to the same concentrations of rGO15 and rGO30. 

These results show that, in general, GO nanostructures were incorporated by macrophages in greater quantity than those of reduced GO and that the main differences in the cellular incorporation of these nanostructures by RAW-264.7 macrophages were observed at the highest dose (10 µg/mL) evaluated in this study. Cellular uptake of a great variety of nanomaterials is known to be highly dependent on their different physicochemical characteristics such as their lateral dimension, oxidation level, and surface functional groups, as well as on their concentration, purity, and shape, among other factors [40]. Regarding GBM, their internalization into cells is strongly influenced by particle size and surface chemistry [41]. The influence of the thickness of GO on cellular internalization is an open debate. While a few studies reported that GO lateral size is a prime factor at determining cellular uptake, with large lateral size preventing cellular uptake [42], other researches have demonstrated that the saturated uptake amount of GO sheets after 24 h did not vary with the lateral dimension (2 and 350 nm), and identical accumulation occurred in primary macrophages when exposed to doses (2 and 6 µg/mL) similar to those in the current study [43]. Here, although the overall lateral size of the rGO sheets decreased upon reduction, it concomitantly underwent aggregation. Reduction-induced aggregation may have a role in the observed decrease in cellular uptake activity when reducing GO. This has been shown for Fe^2+^-reduced GO on a murine macrophage cell line [12]. Additionally, it is expected that at a higher dose, aggregation would be more pronounced, consequently reducing internalization, which was observed here. Another factor that may account for the decreased internalization is related to protein adsorption. Vacuum-assisted thermal reduction induces expansion of the GO sheets, along with an increase in surface area [37]. This surface area increase gives more affinity for extracellular proteins, consequently leading to weaker interactions with the cell membrane and lower cellular uptake. Our XPS analysis revealed, besides increased surface area, that the thermal treatment led to an increase in carboxyl groups, representing a much stronger hydrogen bonding moiety than C-O groups, ensuring stronger hydrogen bonding formation between proteins [12]. The internalization of GBM is also related to the cell type. It has been reported that while GO was internalized by HepG2 cells, by contrast, rGO, which is more hydrophobic than GO, was found to be mostly adsorbed on the cell surface [44]. In addition, it has been demonstrated that different cell types can effectively uptake both GO and rGO nanosheets by different endocytic mechanisms [45,46].

**Figure 5:**
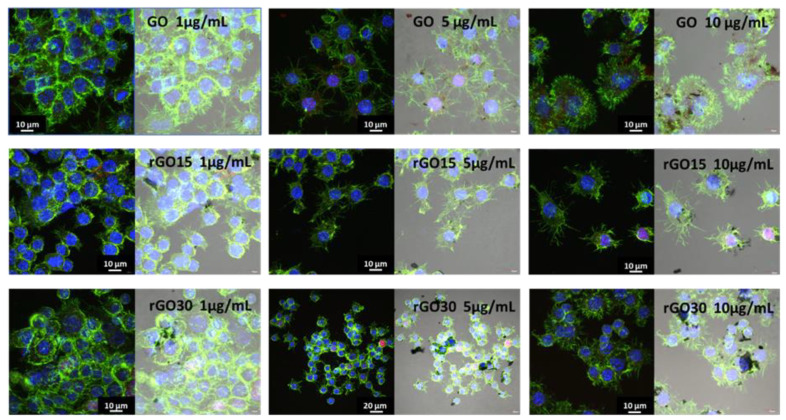
Cell uptake of GO, rGO15, and rGO30 by RAW-264.7 macrophages after 24 h of treatment with 1, 5, and 10 μg/mL, evaluated by confocal and phase contrast microscopy.

In this work, morphological studies of macrophages after GO, rGO15, and rGO30 uptake were carried out by confocal and phase contrast microscopy. Figure 5 shows the confocal images of RAW-264.7 macrophages with their cytoskeleton intact after 24 h of treatment with 1, 5, and 10 μg/mL of these three nanostructures. GO, rGO15, and rGO30 appear as black deposits inside the cells observed by phase contrast microscopy. Adverse effects of GO on murine peritoneal macrophages have been observed by other authors due to the accumulation of this nanomaterial in macrophage lysosomes, leading to lysosome membrane destabilization, autophagosome accumulation, and reduced autophagic degradation [47]. We have evaluated different parameters (included in the following sections) related to the specific function of macrophages to know if the incorporation of these nanostructures induced oxidative stress and promoted a possible inflammatory response.

### 2.3. Intracellular Reactive Oxygen Species (ROS) Content of RAW-264.7 Macrophages after GO, rGO15, and rGO30 Uptake

**Figure 6:**
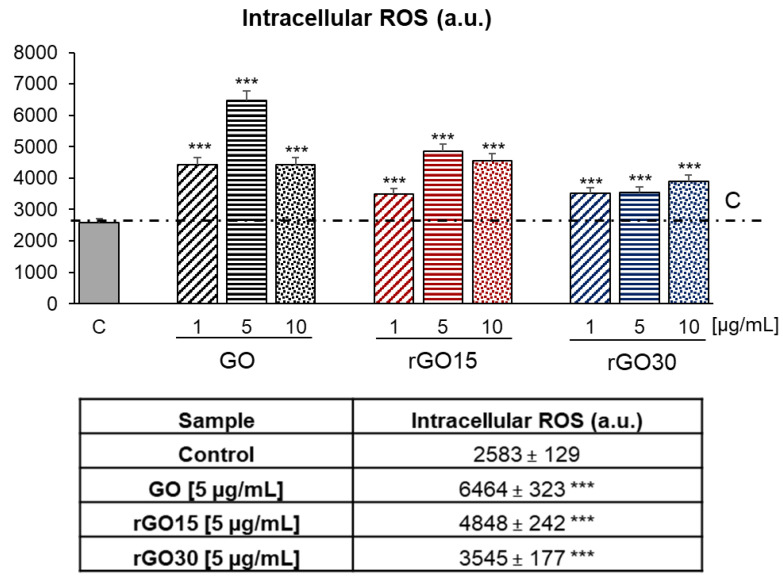
Intracellular content of reactive oxygen species (ROS) of RAW-264.7 macrophages after 24 h of treatment with 1, 5, and 10 μg/mL of GO, rGO15, and rGO30, evaluated by flow cytometry. The table shows the data obtained with the dose of 5 μg/mL of the three nanostructures compared to the value obtained with control macrophages in the absence of material. Statistical significance: *** *p* < 0.005 (compared to control macrophages).

In recent years, numerous GBMs have been developed with the aim of decreasing their toxicity and improving their biocompatibility for use in biomedical applications. Different experimental models have been used in vitro to study the cellular response to graphene and its derivatives, and numerous articles have been published related to the interaction with the components of the immune system [48]. Macrophages represent one of the most useful experimental models, as they are directly involved in the innate immune response and in the uptake of nanoparticles for their elimination from the organism. Concerning the possible adverse effects of these GBMs, several studies propose oxidative stress, mediated by ROS production, as a key mechanism involved in their cytotoxicity [49]. It results from an imbalance between excessive generation of ROS and the limited antioxidant defense capacity of cells, thereby leading to adverse biological effects such as membrane lipid peroxidation, protein denaturation, mitochondrial dysfunction, and DNA damage. Moreover, ROS generation by living cells in response to these kinds of nanomaterials depends greatly on their layer number, lateral size, purity, dose, surface chemistry, dispersibility, and hydrophilicity [45]. Stimulated ROS production was originally described in phagocytic cells such as neutrophils and macrophages. Macrophages are one of the most versatile types of immune cells carrying out a variety of key functions, including phagocytosis of apoptotic cells, bacteria, and viruses, production of reactive nitrogen and oxygen species, antigen processing and presentation, and cytokine and chemokine production. These immune cells also play a central role in directing the host response to implanted biomaterials, including the inflammatory and reparative response related to the M1 and M2 phenotypes, respectively [50,51]. Moreover, it is well-known that M1 proinflammatory macrophages produce and secrete higher ROS levels than M2 reparative cells [52], inducing damage to neighboring cells and promoting the proinflammatory response. Thus, the effects of 1, 5, and 10 μg/mL of GO, rGO15, and rGO30 on the intracellular content of reactive oxygen species (ROS) of RAW-264.7 macrophages were evaluated in the present study by flow cytometry after 24 h of treatment with these nanostructures. Figure 6 shows that GO treatment induced significant increases of intracellular macrophage ROS at all assayed doses, obtaining the most pronounced effect with 5 μg/mL. On the other hand, rGO15 and rGO30 produced a significant elevation of intracellular ROS levels, but less prominent than that observed with GO. These results demonstrate that the GO reduction process employed in this work through a vacuum-assisted thermal treatment of the GO sheets at 200 °C improved its biocompatibility by decreasing its ability to induce oxidative stress. Since the most pronounced effect produced by GO was obtained with 5 μg/mL, and to demonstrate more clearly the benefit of GO reduction, the data of the three nanostructures obtained with the dose of 5 μg/mL are shown in the table included in Figure 6, compared to the value obtained with control macrophages in the absence of material. These results show a progressive decrease of ROS content as the reduction process increased. 

In this context, recent studies indicate the absence of rGO (50 μg/mL) cytotoxicity in HepG2 cells when it is obtained by reduction of GO with hydrazine hydrate. This rGO (25 μg/mL) also showed a protective role against the oxidative stress and toxic effects induced by Cd (2 μg/mL) in this hepatic cell line [53].

### 2.4. CD80 Expression by Macrophages after GO, rGO15, and rGO30 Uptake

**Figure 7:**
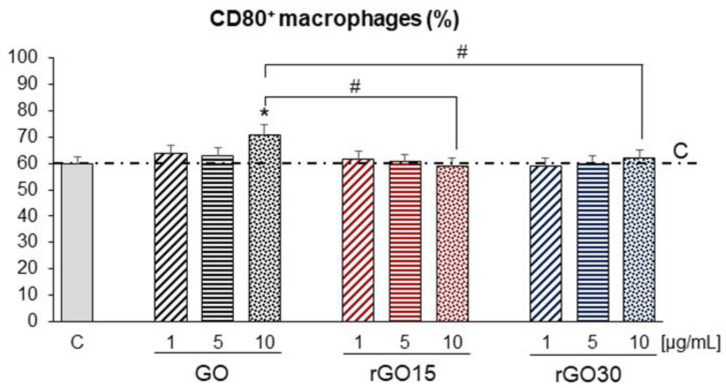
Percentage of the population of CD80^+^ RAW-264.7 macrophages after 24 h of treatment with 1, 5, and 10 μg/mL of GO, rGO15, and rGO30. Statistical significance: * *p* < 0.05 (compared to control macrophages, horizontal line C), # *p* < 0.05 (comparison between nanostructures at the same concentration).

Numerous studies have shown the regulatory role of ROS on the phagocytosis function of macrophages and on their polarization towards M1 or M2 phenotypes, evidencing a dual role in the progression or healing of different diseases. M1 proinflammatory macrophages, also known as classically activated macrophages, are critical for host protection against viruses and intracellular bacteria during acute infections and are involved in helper T cell (Th1) response [54]. This macrophage phenotype is characterized by TLR-2, TLR-4, CD80, CD86, iNOS, and MHC-II surface phenotypes and release various cytokines and chemokines, including tumor necrosis factor (TNF-α) and interleukin IL-1α, IL-1β, IL-6, IL-12, CXCL9, and CXCL10 [55,56]. In addition, M1 macrophages produce microbicidal reagents, such as nitric oxide (NO) and reactive oxygen species (ROS) [57]. In the present study, to find the effects caused by GO, rGO15, and rGO30 nanostructures on the polarization of macrophages towards this M1 phenotype, we have studied different specific markers. In particular, we have evaluated the expression of the CD80 costimulatory molecule on the macrophage surface (CD80^+^ macrophages) and macrophage secretion of proinflammatory cytokines such as TNF-α and IL-6 after GO, rGO15, and rGO30 uptake. Figure 7 displays the CD80^+^ macrophages population percentage after 24 h of treatment with 1, 5, and 10 μg/mL of GO, rGO15, and rGO30. The results evidence a significant increase in the CD80+ macrophages population percentage after exposure to the highest dose of GO (10 µg/mL), compared to control macrophages. However, this effect was not observed after exposure to rGO15 and rGO30 at the highest concentration studied. This data shows that, when using particles, thermal reduction as short as 15 min was enough to mitigate the increase in the population of CD80^+^ when exposed to higher doses of graphene oxide-based particles. 

### 2.5. Detection of TNF-α and IL-6 as Inflammatory Cytokines

**Figure 8:**
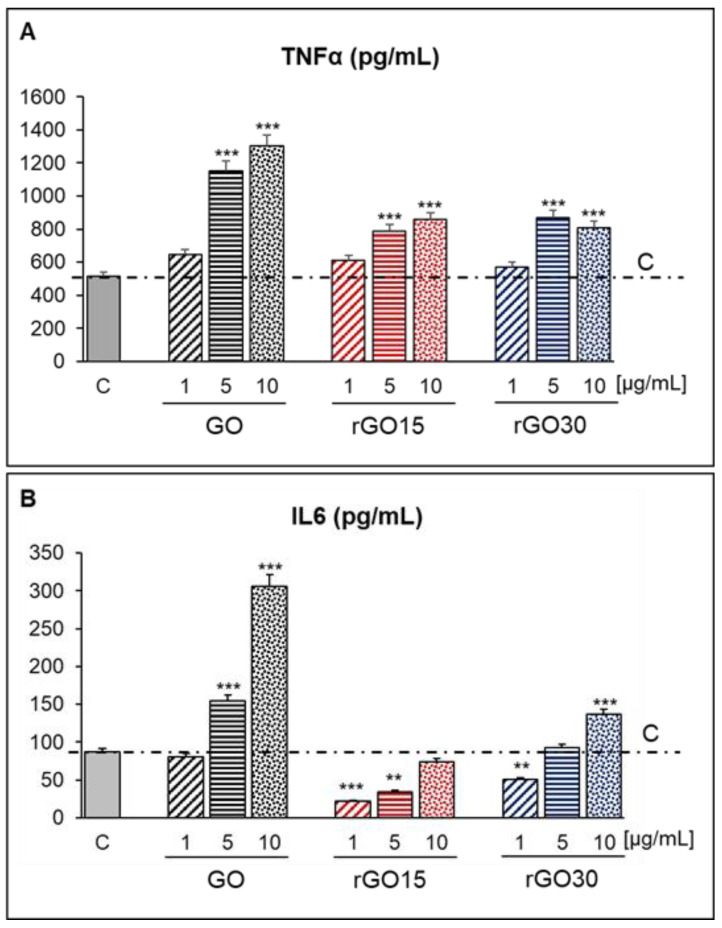
TNF-α (**A**) and IL-6 levels (**B**) (pg/mL) released by RAW-264.7 macrophages after 24 h of treatment with 1, 5, and 10 μg/mL of GO, rGO15, and rGO30 nanostructures, evaluated by ELISA. Statistical significance: ** *p* < 0.01, *** *p* < 0.005 (compared to control macrophages, horizontal line C).

TNF-α and IL-6 are two of the proinflammatory cytokines mainly produced by macrophages polarized towards the M1 phenotype [58]. For this reason, the TNF-α and IL-6 levels released by macrophages after exposure to 1, 5, and 10 µg/mL of GO, rGO15, and rGO30 were evaluated in the present study. Figure 8 clearly shows significant increases in the TNF-α levels secreted by macrophages cultured with 5 and 10 μg/mL of GO, rGO15, and rGO30 compared to those of control macrophages. This effect was more pronounced with GO than with rGO15 or rGO30, showing very high TNF-α secretion induced by GO in a dose-dependent manner. Regarding IL-6, a dose-dependent increase of this cytokine was also detected in the culture medium after treatment with 5 and 10 μg/mL of GO. However, lower levels of IL-6 than that in controls were obtained with the three tested doses of rGO15 (1, 5, and 10 µg/mL), and only 10 µg/mL of rGO30 produced a significant increase of IL-6 compared to that of control macrophages. In this context, when other authors evaluated the effects of 2 μm and 350 nm GO particles on the production of different inflammatory cytokines (IL-6, IL-10, IL-12, TNF-α, MCP-1, IFN-γ) in macrophages, it was shown that the secretion of these mediators was highly dependent on the GO dosage, particularly for the 2 μm GO particles [43]. In thermally reduced rGO microfibers, we previously observed a decrease of TNF-α and IL-6 after 24 h of culturing [26]. Here, the cells were subjected to a distribution of GO sheets rather than contact with bulk fibers and showed higher reactivity to the reduced sheets than to the bulk fibers. This was more evident for a higher concentration of GBM material. Still, thermal reduction as short as 15 min exhibited a significant improvement in terms of decreasing the proinflammatory cues. Our results demonstrate that GO reduction reduced its ability to induce the synthesis and secretion of proinflammatory cytokines, significantly improving its biocompatibility.

GBM shows great promise for biomedical applications and can be designed with different configurations such as nanosheets, nanoparticles, 2D films, and 3D scaffolds [26,34,59]. The GO and rGO nanostructures evaluated in this study are potentially useful for the preparation of scaffolds for tissue regeneration after assessing the effects that these components could have if they are released during implant degradation once they have been introduced into the body. Thus, when biomedical engineers design graphene-based scaffolds, the aspects related to the targeted biomedical scenario, such as scaffold biodegradation and its kinetics, should be carefully probed and understood to avoid an immune response and allow us to fully exploit the potential of GBM. In this context, numerous in vitro and in vivo studies with rGO-prepared scaffolds have shown promising results for the regeneration of different tissues [7,60,61,62,63]. 

The knowledge of the response of macrophages to GBM is particularly important because these cells are responsible for innate immunity [20] and play a key role in the processing of nanomaterials [38]. In this sense, the possibility of modulating macrophage polarization towards a proinflammatory or reparative phenotype with biomaterials is considered a promising strategy to control inflammatory processes and tissue regeneration at the implant site [18]. In this work, we have evaluated different aspects related to the macrophage response to GO, rGO15, and rGO30, evidencing an active and dose-dependent incorporation of these nanostructures by this cell type without inducing the expression of the proinflammatory marker CD80. On the other hand, the rGO-treated macrophages produced lower amounts of reactive oxygen species and proinflammatory cytokines than GO-treated cells, indicating the benefits of the reduction process of this nanomaterial and further supporting the use of rGO in the preparation of novel scaffolds.

## 3. Materials and Methods

### 3.1. Preparation of GO, rGO15, and rGO30 Nanostructures

The GO used in this work was of commercial origin (Graphenea^®^, San Sebastián, Spain). According to the supplier, in its original source (0.4 wt% aqueous solution) it has monolayer content (measured in 0.05 wt%) higher than 95% and particle lateral size lower than 10 µm. GO is prepared from chemical exfoliation of graphite using a strong oxidant and acidic media. Therefore, pH of the original GO solution is between 2.2 and 2.5, which favors nanosheet dispersion in the media. Other than the acidic residues, other chemical moieties like sulfur and manganese are present due to the exfoliation methodology. The presence of these residues, which can be toxic to cells, can be mitigated to some extent by dialysis treatment. Therefore, the commercial GO dispersion was firstly dialyzed with distilled water that replaced daily for a week. Afterwards, this solution was freeze-dried to obtain chemical-free GO sheets in a Teslar lyoQuest HT-40 freeze-drier ( Beijer Electronics Products AB, Sweden). However, it must be considered that this process may result in the agglomeration of GO nanosheets due to rinsing at the pH of the medium and consequent manipulations, such as freeze-drying. Even so, we considered this procedure important to remove chemical impurities that may be related to the often-proclaimed toxic effects of GO on cells [64].

### 3.2. Morphological and Structural Characterization of GO, rGO15, and rGO30 Nanostructures

Dispersions of GO, rGO15, and rGO30 at 0.5 mg/mL were spin coated on glass coverslips at 800 rpm to analyze the morphology via atomic force microscopy (Bruker Multimode instrument (Bruker Nano Surfaces, Santa Barbara) with a Nanoscope (IV) MMAFM-2 unit) with a conductive Si cantilever (Nanosensors, force constant 15 N/m, Neuchatel, Switzerland).

Structural characterization of GO before and after the 15- and 30-min thermal treatments was performed by X-ray diffraction (XRD) and X-ray photoelectron spectroscopy (XPS). XRD spectra were acquired from 5 to 80° at a scanning speed of 1°/min in a Rigaku SmartLab diffractometer (Rigaku Corporation, Japan) using Cu Kα radiation (λ = 1.5406 Å). 

XPS (with a hemispherical electron energy analyzer SPECS Phoibos 150 (Berlin, Germany) and a monochromatic Al Kα (1486.74 eV) X-ray source) was performed in an ultra-high vacuum system (with a base pressure of 2 × 10^−8^ Pa) at a normal emission take-off angle and 20 eV pass-energy. 

### 3.3. Culture of RAW-264.7 Macrophages for Treatment with GO, rGO15, and rGO30

RAW-264.7 macrophages were seeded with cell density of 1 × 10^5^ cells/mL in Dulbecco’s Modified Eagle Medium (DMEM, Gibco BRL, United Kingdom) supplemented with 10% fetal bovine serum (FBS, Gibco BRL, United Kingdom), 1 mM L-glutamine (BioWhittaker Europe, Verviers, Belgium), 800 μg/mL penicillin (BioWhittaker Europe, Verviers, Belgium), and 800 μg/mL streptomycin (BioWhittaker Europe, Verviers, Belgium) in a 5% CO_2_ humidified atmosphere at 37 °C for 24 h. Then, the culture medium was replaced by fresh medium containing GO, rGO15, and rGO30 at different concentrations (1, 5, and 10 μg/mL) that was previously sonicated for 5 min to homogenize the mixture. These doses were chosen based on previous studies with macrophage cultures as an in vitro experimental model [19,65] and considering recent toxicity in vivo studies in mice [66]. After culturing the cells for 24 h under these conditions, the macrophages were first washed with phosphate-buffered saline (PBS, Sigma-Aldrich) to remove the nonincorporated nanomaterials and then detached with a scraper before analyzing all cell response-specific studies. Control samples corresponding to macrophages cultured in the absence of nanomaterials were included in all the assays.

### 3.4. Uptake of GO, rGO15, and rGO30 by RAW-264.7 Macrophages Evaluated by Flow Cytometry and Confocal and Phase Contrast Microscopy

The incorporation of GO, rGO15, and rGO30 by RAW-264.7 macrophages after 24 h of treatment was quantified by flow cytometry analyzing 90° light scatter (side scatter, SSC) that allows to evaluate nanomaterial uptake by mammalian cells [67,68]. The SSC parameter is proportional to the intracellular complexity determined in part by the cellular cytoplasm, mitochondria, and pinocytic vesicles [39]. The conditions for data acquisition and analysis were established using negative and positive controls with the CellQuest Program of Becton Dickinson, and these conditions were maintained in all the experiments. Each experiment was carried out three times, and single representative experiments are displayed. For statistical significance, at least 10,000 cells were analyzed by flow cytometry in each sample. The incorporation of GO, rGO15, and rGO30 by macrophages was observed by confocal and phase contrast microscopy as in previous studies [19]. For these confocal and phase contrast microscopy studies, RAW-264.7 macrophages were cultured on circular glass coverslips under the above-mentioned cell culture conditions. Cells were fixed with 3.7% paraformaldehyde (Sigma-Aldrich Corporation, St. Louis, MO, USA) in PBS for 10 min, washed with PBS, and permeabilized with 0.1% Triton X-100 (Sigma-Aldrich Corporation, St. Louis, MO, USA) for 5 min. The samples were then washed with PBS and preincubated with PBS containing 1% BSA (Sigma-Aldrich Corporation, St. Louis, MO, USA) for 30 min to prevent nonspecific binding. The samples were incubated in 1 mL of staining buffer with PE-conjugated anti-mouse CD80 antibody (2.5 µg/mL, BioLegend, San Diego, CA, USA) for 30 min at 4 °C in the dark. The samples were then washed with PBS, and the cell nuclei were stained with DAPI (4′-6-diamidino-2′-phenylindole, 3 μM in PBS, Molecular Probes, Eugene, OR, USA) for 5 min. The samples were examined in a LEICA SP2 Confocal Laser Scanning Microscope. Fluorescence PE was excited at 488 nm, and the emitted fluorescence was measured at 575–675 nm. DAPI fluorescence was excited at 405 nm and measured at 420–480 nm. 

### 3.5. Measurement of Intracellular Reactive Oxygen Species (ROS) Content of Macrophages by Flow Cytometry after GO, rGO15, and rGO30 Uptake

After exposure to GO, rGO15, and rGO30 for 24 h, RAW-264.7 macrophages were detached, and cell suspensions were incubated with 10 μM of 2’,7’-dichlorodihydro fluorescein diacetate (DCF-H2-DA, Serva, Heidelberg, Germany) for 45 min at 37 °C. The nonfluorescent DCF-H2-DA transforms into 2’,7’-dichlorofluorescein (DCF) after hydrolysis by cellular esterases and oxidation by ROS. When DCF is excited at 488 nm emission wavelengths, it emits green fluorescence that can be detected at 525 nm. DCF fluorescence was measured in a FACScalibur Becton Dickinson flow cytometer with a 530/30 filter, exciting the sample at 488 nm. For statistical significance, at least 10,000 cells were analyzed by flow cytometry in each sample.

### 3.6. Detection of Macrophage M1 Proinflammatory Phenotype by Flow Cytometry after GO, rGO15, and rGO30 Uptake

The expression of CD80 was used as a specific marker to identify M1 macrophages [69] and quantified by flow cytometry after exposure of RAW-264.7 macrophages to GO, rGO15, and rGO30. Before immunostaining, the cells were detached and incubated in 45 µL of staining buffer (PBS Thermo Fisher Scientific Madrid, Spain, 2.5% FBS Gibco BRL United Kingdom Gibco, and 0.1% sodium azide, Sigma-Aldrich Corporation, St. Louis, MO, USA) with 5 µL of normal mouse serum inactivated for 15 min at 4 °C in order to block the Fc receptors on the macrophage plasma membrane and to prevent nonspecific binding of the primary antibody. Then, the cells were incubated with phycoerythrin (PE)-conjugated anti-mouse CD80 antibody (2.5 µg/mL, BioLegend, San Diego, CA, USA) for 30 min in the dark. Labeled macrophages were then analyzed using a FACSCalibur flow cytometer. The fluorescence was excited at 488 nm and measured at 585/42 nm. The conditions for data acquisition and analysis were established using negative and positive controls with the CellQuest Program of Becton Dickinson, and these conditions were maintained in all the experiments. Each experiment was carried out three times, and single representative experiments are displayed. For statistical significance, at least 10,000 cells were analyzed in each sample.

### 3.7. Detection of TNF-α and IL-6 as Inflammatory Cytokines

The amount of TNF-α and IL-6 secreted by RAW-264.7 macrophages under the different conditions was quantified in the culture medium by enzyme-linked immunosorbent assay (ELISA, Gen-Probe, Diaclone, Besançon, France) according to the manufacturer’s instructions.

### 3.8. Statistics

Data are expressed as means ± standard deviations of a representative of three experiments carried out in triplicate. Statistical analysis was performed using the Statistical Package for the Social Sciences (SPSS) version 22 software. Statistical comparisons were made by analysis of variance (ANOVA). Scheffé test was used for post hoc evaluations of differences among groups. In all the statistical evaluations, *p* < 0.05 was considered as statistically significant.

## 4. Conclusions

Our comparative study with RAW-264.7 macrophages that evaluated specific parameters of their in vitro response to different graphene-based nanomaterials has demonstrated the benefits of GO reduction by vacuum-assisted thermal treatment at 200 °C to obtain nanostructures with higher biocompatibility, improving their potential for the preparation of 3D scaffolds that are able to trigger the appropriate immune response for tissue regeneration.

## Data Availability

Data is contained within the article.
